# Effectiveness of nonclinic-based dual-task training on physical and cognitive functions and activities of daily living in older adults with cognitive decline: A systematic review and meta-analysis

**DOI:** 10.1371/journal.pdig.0001571

**Published:** 2026-08-06

**Authors:** Jeerati Rattanatreyanupab, Htet Htet Hnin, Theerapol Witthiwej, Vimonwan Hiengkaew, Pagamas Piriyaprasarth, Yasutaka Nikaido, Ammarin Thakkinstian, Kunlawat Thadanipon, Saharat Liampeng, Sunee Bovonsunthonchai

**Affiliations:** 1 Faculty of Physical Therapy, Mahidol University, Nakhon Pathom, Thailand; 2 Division of Neurosurgery, Department of Surgery, Faculty of Medicine Siriraj Hospital, Mahidol University, Bangkok, Thailand; 3 Clinical Department of Rehabilitation, Osaka Medical and Pharmaceutical University Hospital, Osaka, Japan; 4 Department of Clinical Epidemiology and Biostatistics, Faculty of Medicine Ramathibodi Hospital, Mahidol University, Bangkok, Thailand; University of Salford, UNITED KINGDOM OF GREAT BRITAIN AND NORTHERN IRELAND

## Abstract

Older adults with cognitive decline commonly demonstrate deficits in memory, attention, and problem-solving, leading to reduced ability to perform activities of daily living (ADL). Nonclinic-based dual-task training (DTT), delivered at home, in community, or via telerehabilitation, aligns with digital health strategies to improve accessibility and continuity of care for older adults with cognitive decline. This review examined the effects of such intervention on gait, functional mobility, cognitive performance, and ADL, as well as adverse events, feasibility, acceptability, and caregiver burden. Six electronic databases, including PubMed, Embase, CINAHL, CENTRAL, IEEE Xplore, and ProQuest, were systematically searched. Eligible studies were randomized controlled trials (RCTs) investigating the effectiveness of nonclinic-based DTT on variables in older adults with cognitive decline. The risk of bias was assessed using the revised Cochrane risk-of-bias tool for randomized trials version 2 (RoB 2). Data were synthesized using pairwise meta-analysis to compare the DTT with active or passive controls, and network meta-analysis to compare and rank treatment approaches. Twenty-three RCTs involving 1,674 participants were included. The DTT demonstrated significantly greater improvements than the active control in functional mobility (SMD = 0.49; 95% CI, 0.08–0.89; *p* = 0.019) and global cognition (SMD = 0.32; 95% CI, 0.10–0.55; *p* = 0.005). Compared with the passive control, the DTT also showed greater gains in gait speed (USMD = 0.05 m/s; 95% CI, 0.02–0.08; *p* = 0.001) and global cognition (SMD = 0.80; 95% CI, 0.37–1.23; *p* < 0.001). Only minimal adverse effects were reported, while the mean adherence rate across studies was 80.82%, despite identified barriers including technical difficulties, lack of motivation, and caregiver burden. These findings support its feasibility and effectiveness as a complementary approach to conventional interventions, offering a practical alternative that may reduce the reliance on frequent clinic visits and facilitate greater functional independence.

## Author summary

Nonclinic-based interventions, particularly home-based rehabilitation supported by digital health technologies, are increasingly recognized as an effective alternative or complement to traditional clinical care. Approaches such as exercise-based videogames, virtual reality, and telerehabilitation enable individuals to engage in intervention at home while receiving guidance, feedback, and follow-up from clinicians. For older adults with cognitive decline, including mild cognitive impairment, Alzheimer’s disease, and dementia, exercise is strongly recommended to prevent further functional decline and to maintain or improve physical and cognitive abilities. Dual-task training combines physical and cognitive activities, such as walking while performing simple mental calculation tasks. This approach is increasingly used to help older adults maintain or improve both physical and cognitive abilities. It is widely used in clinics and has been proven to effectively challenge integrated cognitive and motor functions more efficiently than single-task training. In this review, we synthesized evidence on nonclinic-based dual-task training in older adults with cognitive decline. The findings suggest that programs combining exercise with activities that keep the mind active can improve both physical health and thinking skills. These programs can be done at home or in the community, making them a convenient option for people who cannot attend programs at hospitals or clinics.

## Introduction

Cognitive impairment serves as an umbrella term for a broad spectrum of cognitive decline, which commonly occurs in older adults, with global prevalence from 5.1% to 41.0% and incidence from 22 to 77.8 per 1,000 person-years [1]. Within the clinical spectrum, mild cognitive impairment (MCI) represents a specific, early stage of cognitive decline characterized by measurable changes in memory or thinking, though independence in daily activities remained. Individuals with MCI are at high risk of progressing to dementia, particularly Alzheimer’s disease (AD) [2,3], or returning to normal cognition [4]. However, varying degrees of cognitive impairment are also commonly observed across a wide range of neurological disorders, such as stroke, Parkinson’s disease (PD), and multiple sclerosis (MS) [5–7].

Individuals with MCI typically experience short-term memory loss, reduced learning ability, poor attention, and difficulties in problem-solving, all of which interfere with daily functioning [1]. Emerging evidence underscores the bidirectional relationship between cognitive impairment and physical decline [8,9]. Individuals with MCI and AD often present with reduced muscle strength, slow gait speed, and impaired balance [10–12], indicating that cognitive and physical functions tend to deteriorate concurrently in these populations.

Exercise is a safe, low-cost nonpharmacological therapy that improves muscle strength, balance, and cognitive performance partly through neurogenesis, cardiovascular health, and enhances mitochondrial stability [13,14]. The cognitive effect of exercise is linked to neural plasticity, brain-derived neurotrophic factor (BDNF) production, and improved energy metabolism [15,16]. It also activates organ-brain signaling by releasing cytokines linked to cognitive function [16]. For individuals with MCI or dementia, several exercise modalities have been shown to be effective, including aerobic training, resistance training, mind-body exercise, and multicomponent programs [17]. Existing evidence indicates that resistance training may help slow cognitive decline, while multicomponent exercise appears beneficial for improving global cognition and executive function [18].

Dual-task training (DTT), defined as the simultaneous performance of two tasks, typically integrates motor and/or cognitive components. It includes motor-motor tasks (e.g., holding a glass of water while walking) and motor-cognitive tasks (e.g., counting backward while walking), the latter commonly used to address physical and cognitive decline in older adults [19]. DTT outperforms single-task training (STT) in enhancing cognitive and physical functions in stroke [20], PD [21], and cognitive impairment [22]. Exergames, a technology-based form of DTT that combines physical and cognitive tasks, may improve attention, memory, muscle strength, and cardiorespiratory endurance in older adults and individuals with cognitive decline [23–25]. With delivery platforms such as motion capture cameras, step mats, or virtual reality (VR), exergames offer a convenient, home-based solution for advancing physical and cognitive functions, allowing training to be delivered at home or in community settings, reducing dependence on clinic visits [26,27]. The shift toward delivering rehabilitation outside traditional clinical environments has gained momentum, particularly for older adults with mobility limitations or limited access to care. Home-based or telerehabilitation approaches offer practical solutions for implementing DTT and exergames interventions using communication technologies such as videoconferencing [28,29].

Although evidence supporting DTT and exergames is increasing, most reviews have focused on clinic-based interventions or specific neurological conditions [30–34]. Only one recent systematic review assessed DTT in individuals with cognitive decline [35], but it did not specifically address nonclinic-based delivery. To fill this gap, this systematic review aimed to investigate the effectiveness of nonclinic-based DTT, including home-based, telerehabilitation-delivered DTT, and exergames, on physical function, cognitive function, and activities of daily living (ADL) in older adults with cognitive decline.

## Materials and methods

This systematic review was conducted following the Preferred Reporting Items for Systematic Review and Meta-Analyses (PRISMA) guidelines [36], the details of which are described in S1 Checklist **PRISMA Checklist**, and was registered at the International Prospective Register of Systematic Reviews (PROSPERO) (CRD420250649904).

### Data sources and search strategy

Relevant studies were identified via the following electronic databases: PubMed, Embase, Cumulative Index to Nursing and Allied Health Literature (CINAHL), Cochrane Central Register of Controlled Trials (CENTRAL), Institute of Electrical and Electronics Engineers (IEEE) Xplore, and ProQuest (theses and dissertations). The search terms and strategies were constructed based on patient, intervention, comparator, and outcome framework. To ensure a comprehensive and methodologically rigorous search strategy, relevant Medical Subject Headings (MeSH) and comprehensive free-text synonyms for each key concept were identified. Preliminary searches were conducted to assess the retrieval performance of individual search terms and to identify redundant or non-contributory keywords. The search strategy was iteratively refined by combining synonymous terms using the Boolean operator “OR” and integrating distinct conceptual domains using the operator “AND.” This process was repeated until an optimal search sensitivity was achieved. The final search strategies for all databases are presented in the **S1 Table**. The last search was conducted on 10 March 2025.

### Study selection

All identified articles were imported into the Covidence platform, where duplicates were automatically removed. No language restrictions were applied during the search and study selection process. The remaining articles were then screened according to the following eligibility criteria: (1) RCT study design; (2) published between 2014 and 2025; (3) adults (≥ 60 years old) diagnosed with any of cognitive impairment conditions, e.g., MCI, dementia, or AD; (4) had intervention as DTT or exergames via home-based or telerehabilitation approaches; (5) had any type of active (e.g., STT or exercise education) or passive controls (i.e., no intervention, general health education, usual care, or stretching); and (6) had any of these outcomes: gait speed, functional mobility, global cognition, and ADL. Studies were excluded if: (1) unavailable full-text article, (2) study protocols or abstracts, or (3) duplicate publications of the same study.

Two independent reviewers (JR and HHH) conducted the study selection process. Titles and abstracts were initially screened to identify potentially relevant studies. The full texts were subsequently reviewed to confirm their final inclusion. To evaluate the agreement between the two reviewers during the selection process, Cohen’s kappa statistic was used at both the title/abstract and full-text selection stages. Discrepancies were resolved through discussion, and if consensus could not be reached, a third reviewer (SB) made the final decision.

### Interventions

The intervention arms in this review consisted of three groups: DTT, active control, and passive control. The intervention of interest was DTT delivered via home-based, community-based, or telerehabilitation approach, with exergame classified under the same DTT category.

DTT is defined as an intervention in which participants perform two or more tasks simultaneously. These tasks could involve either motor-motor components (e.g., walking while carrying a glass of water) or motor-cognitive components (e.g., walking while counting backward). An exergame is defined as video game-based interventions that combine physical activity with cognitive engagement. Examples include games displayed on a television or monitor, floor-projected games requiring stepping movements, games combined with exercise equipment, and games delivered via immersive VR technology. Home-based approaches refer to interventions delivered in the participant’s home or community settings rather than in healthcare facilities such as hospitals or clinics. Telerehabilitation refers to interventions delivered remotely through communication technologies, such as telephone or video conferencing, that connect therapists and participants.

### Outcomes of interest

The primary outcomes were physical functions including gait speed (assessed under single-task conditions), functional mobility [e.g., Timed Up and Go (TUG), chair stand, or sit-to-stand test], cognitive function [e.g., Mini-Mental State Examination (MMSE), Montreal Cognitive Assessment (MoCA), or other tools reflecting global cognition], and ADL measures [e.g., Barthel Index, Katz Index of Independence in ADL, or Functional Independence Measure]. The secondary outcome measures were adverse events (type and incidence), feasibility, acceptability, and caregiver burden.

### Data extraction and literature quality assessment

Data were independently extracted by two reviewers (JR and HHH) using a standardized data extraction form. After extractions, two data sets were then cross-validated, and discrepancies were resolved through discussion and consensus. When consensus could not be reached, a third reviewer (SB) acted as an adjudicator and made the final decision. Data were extracted on the following aspects: author, publication year, region, and participant characteristics (sample size, age, sex, type of cognitive impairment, and baseline cognitive scores). Details of the intervention and comparator groups, including the type, duration, and frequency of the intervention, were recorded. The outcome measures were categorized into four outcomes: gait speed, functional mobility, global cognition, and ADL. The extracted data used for the meta-analysis are provided in **S1 Data**.

For studies reporting baseline cognitive performance via the MoCA, scores were converted to equivalent MMSE scores [37]. Because the crosswalk table provides estimated scores as integers only, a linear interpolation method was applied to derive values with decimal precision*.* This procedure ensures a consistent measure of the baseline cognitive score across studies, facilitating accurate inclusion in the meta-regression analyses.

The methodological quality of the included studies was assessed by the two reviewers using the revised Cochrane risk-of-bias tool for randomized trials version 2 (RoB 2) [38]. The RoB 2 tool evaluates five domains: the randomization process, deviations from intended interventions, missing outcome data, measurement of the outcome, and selection of the reported result. Each domain was rated as “low risk,” “some concerns,” or “high risk” of bias, in accordance with the tool’s guidelines [38].

### Data synthesis and analysis

A pairwise meta-analysis was conducted when at least three studies reported the same outcome and comparator. Unstandardized mean differences were estimated and pooled across studies if the outcomes were measured using the same scale; otherwise, the standardized mean differences (Hedges’ *g*) were applied. A random-effect model was used when heterogeneity was present (*I*^2^ ≥ 25% or Cochran’s Q test *p* < 0.1); otherwise, a fixed-effects model was used. Forest plots were used to visualize the effect of each study and the combined overall effect in each pair.

Meta-regression was performed to explore potential sources of heterogeneity by regressing effect size on individual covariate one by one, including geographic region, participant characteristics (age, sex, and baseline MMSE score), and details of the intervention and comparator (type, mode, duration, frequency, and total number of weeks of intervention). A covariate was considered influential if it reduced the τ² by at least 50% compared with the empty model.

Subgroup analyses were conducted by prior-defined factors including the type of DTT (DTT or exergames), delivery mode (telerehabilitation or home-/community-based), and severity of cognitive decline (MMSE ≥ 27 normal; 21–26 mild; 15–20 moderate; and < 15 severe) [39]. Additional subgroup analyses were conducted if meta-regression identified potential factors.

A two-stage network meta-analysis was performed as follows: First, a network map was generated to visualize intervention nodes and comparison-pairs across studies. Intervention groups were coded as DTT, active control, and passive control. Second, intervention-effect sizes (by unstandardized mean difference or standardized mean difference where appropriate) were pooled across studies using a multivariate meta-analysis with consistency model, relative intervention-effects were estimated accordingly and presented in the league tables for all comparison pairs. Intervention ranking was assessed using the surface under the cumulative ranking curve analysis (SUCRA). Publication bias was determined using a comparison-adjusted funnel plot and Egger’s test. Consistency assumption was checked using a global Chi-square test, and transitivity assumption was explored by comparing characteristics across comparison-pairs. All the statistical analyses were conducted using Stata/MP version 18.5 (StataCorp LLC, College Station, TX, USA). A p-value less than 0.05 was considered statistically significant, except for heterogeneity (*p* < 0.1).

## Results

### Study selection and characteristics

**Fig 1:**
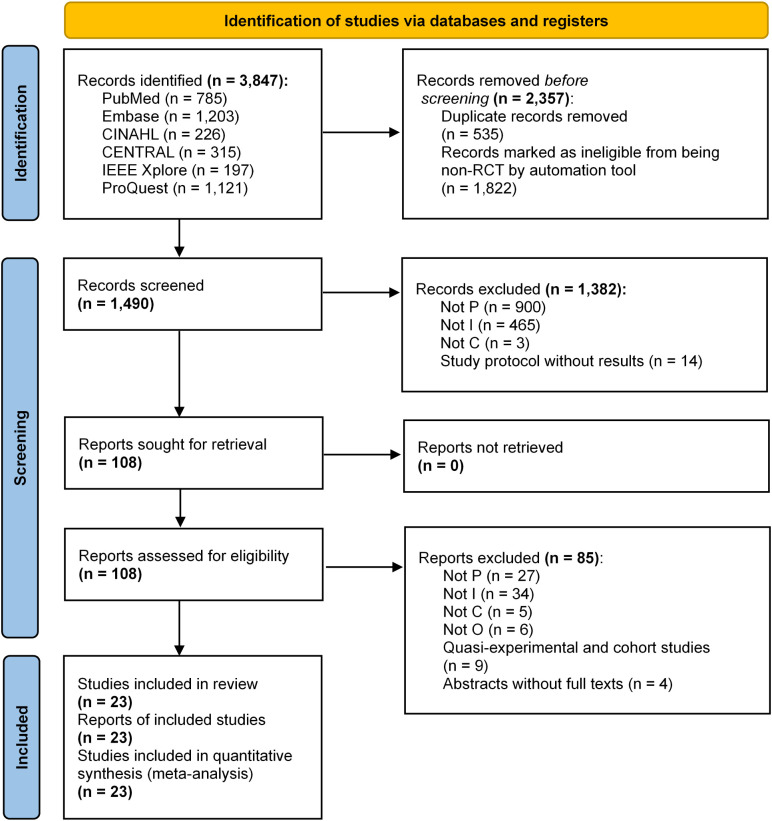
PRISMA flow diagram of study selection.

A total of 3,847 records were initially identified through electronic databases, and 23 studies met the eligibility criteria for inclusion in the analysis [40–62]. Reasons for exclusion are summarized in the PRISMA flow diagram **(Fig 1**), with detailed reasons for exclusion at the title and abstract screening and full-text screening stages are presented in **S2 Table**. The inter-rater agreement between the two independent reviewers, as measured by Cohen’s kappa, was 0.517 for title and abstract screening and 0.516 for full-text screening. Disagreements requiring adjudication by a third author were mainly due to unclear or insufficient reporting in the study descriptions. At the title and abstract stage, these typically involved ambiguity in the reporting of the study population, intervention, or design, resulting in differences in decisions regarding progression to full-text screening. At the full-text stage, disagreements primarily concerned borderline eligibility, particularly in relation to the classification of cognitive status and the adequacy of multi-component interventions in representing the targeted training modality. All discrepancies were resolved through third-author adjudication to ensure consistency in applying the inclusion criteria.

Summary of the study characteristics, participant demographics, interventions, comparators, and outcome measures are presented in **Table 1**. Among the 23 included studies, 1,674 participants were involved. Across the included studies, mean age ranged from 66.57 to 86 years (overall mean age 77.18 years), and the proportion of female participants ranged from 28.13% to 94.44% (overall 61.17%). Studies were conducted across 14 countries, including United States (n = 6), Taiwan (n = 3), China (n = 2), Turkey (n = 2), Hong Kong (n = 1), Spain (n = 1), Netherlands (n = 1), Belgium (n = 1), Switzerland (n = 1), Germany (n = 1), Australia (n = 1), Israel (n = 1), Japan (n = 1), and Korea (n = 1). Interventions included 11 exergames and 12 non-exergame DTT. Delivery modes comprised 16 community-based delivery, 5 tele-rehabilitation, and 2 home-based approaches. Focusing on 18 home- and community-based rehabilitation, six studies integrated games or virtual environments by using screen, projection, or VR headset with exercise equipment such as interactive cycling, interactive resistance exercise, and virtual kayak paddling [40,42,46,52,60,61]. Six studies used commercial consoles and motion capture, such as Nintendo Wii Fit and Microsoft Kinect [44,45,50,51,55,59]. Three studies implemented DTT as an application on computers or tablets with specialized sensors [43,53,57]. Nevertheless, three studies did not use digital tools involving the intervention process; they were only used in evaluation and assessment [47,58,62]. When analyzing intervention techniques, technology-aided interventions build upon the fundamental motor-cognitive dual-task principles used in standard DTT. Traditional interventions typically induce motor-cognitive interference through tasks such as unstable surface training or concurrent cognitive tasks (e.g., verbal or attentional tasks), whereas technology-based approaches, including VR environments, simulate more complex functional contexts that engage visual attention and provide real-time feedback. Overall, both approaches share a common dual-task framework; however, digital interventions may offer greater environmental complexity and task variability, which have been associated with improvements in dual-task gait performance [63]. Regarding intervention delivery, DTT programs were primarily implemented by research personnel or trained instructors following standardized protocols. However, four studies reported individualized one-on-one interventions that were directly prescribed, customized, or supervised by clinicians [41,53–55]. In terms of effectiveness, previous studies of in-person DTT have generally reported beneficial effects on motor and cognitive outcomes, although the magnitude of effects varies depending on intervention dose, task complexity, and target population [64].

**Table 1:** Characteristics of the included studies (n = 23).

Studies	Region	Participants					Intervention		Control	Duration per session, frequency, and cycle	Outcome measures
		Sample size	Mean age (years)	Overall % of females	Mean baseline MMSE	Type of cognitive impairment	Type of intervention	Mode of delivery			
Anderson-Hanley et al., 2018 [40]	United States	IG: 7 CG: 7	IG: 75.4 CG: 80.9	50.00	25.03^a^	MCI	Exergame (Exer-tour: cycling exercise combined with video game)	Community-based	STT	Not specified	TUG
Callisaya et al., 2021 [41]	Australia	IG: 44 CG: 49	IG: 72.9 CG: 72.8	58.06	28.45^a^	MCI	DTT (StandingTall program: balance and strength training combined with cognitive tasks)	Telerehabilitation	General health education	2 hours/week, 8 weeks	Gait speed, 5TSTS
Choi et al., 2019 [42]	Korea	IG: 30 CG: 30	IG: 77.27 CG: 75.37	85.00	24.15^a^	MCI	Exergame (virtual kayak paddling exercise)	Community-based	Exercise education	60 minutes, 2 sessions/week, 6 weeks	TUG, MoCA
Embon-Magal et al., 2022 [43]	Israel	IG: 27 CG: 19	IG: 83.33 CG: 78.67	65.22	21.04^a^	Dementia	DTT (Thinking in Motion (TIM): combined physical and cognitive exercise)	Community-based	STT	40 minutes, 3 sessions/week, 8 weeks	Gait speed, CogniFit computerized neurocognitive battery
Hughes et al., 2014 [44]	United States	IG: 10 CG: 10	IG: 78.5 CG: 76.2	70.00	27.15	MCI	Exergame (interactive video gaming with physical action)	Community-based	Exercise education	90 minutes, 1 session/week, 24 weeks	Gait speed, IADL
Jahouh et al., 2021 [45]	Spain	IG: 40 CG: 40	IG: 85.05 CG: 83.25	56.25	22.19	Dementia	Exergame (Interactive video gaming with physical action)	Community-based	No treatment	45 minutes, 3 sessions/week, 20 sessions	MCE, Barthel index
Kwan et al., 2024 [46]	Hong Kong	IG: 146 CG: 147	IG: 75.2 CG: 73.9	78.16	24.67^a^	MCI	Exergame (virtual reality motor-cognitive training)	Community-based	Usual care	60 minutes, 2 sessions/week, 8 weeks	TUG, MoCA
Lee et al., 2023 [47]	Japan	IG: 140 CG: 140	IG: 76.27 CG: 76.42	39.64	N/A	Dementia	DTT (Cognicise: aerobic exercise combined with cognitive tasks)	Community-based	General health education	90 minutes, 1 sessions/week, 40 weeks	Gait speed, 5TSTS
Li et al., 2021 [48]	United States	IG: 15 CG: 15	IG: 76.13 CG: 76.2	70.00	25.17	MCI	DTT (cognitively-enhanced Tai Ji Quan training)	Telerehabilitation	Stretching	60 minutes, 2 sessions/week, 24 weeks	TUG
Li et al., 2022 [49]	United States	IG: 23 CG1: 22 CG2: 24	IG: 74.4 CG1: 74.5 CG2: 74.9	56.52	26.93	MCI	DTT (cognitively-enhanced Tai Ji Quan training)	Telerehabilitation	STT; Stretching	60 minutes, 2 sessions/week, 16 weeks	MoCA
Liao et al., 2020 [50]	Taiwan	IG: 16 CG: 18	IG: 73.1 CG: 75.5	67.65	27.20	MCI	DTT (combined physical and cognitive exercise)	Community-based	STT	60 minutes, 3 sessions/week, 12 weeks	IADL
Liao et al., 2021 [51]	Taiwan	IG: 25 CG: 21	IG: 79.6 CG: 83.8	67.39	22.91	MCI	DTT (combined physical and cognitive exercise)	Community-based	STT	60 minutes, 3 sessions/week, 12 weeks	MoCA
Liao et al., 2025 [52]	Taiwan	IG: 20 CG: 19	IG: 77.5 CG: 79.3	82.05	24.95	MCI	Exergame (interactive boxing-cycling training combined with cognitive tasks)	Community-based	STT	40 minutes, 1 session/week, 12 weeks	Gait speed, MoCA
Manser & de Bruin, 2024 [53]	Switzerland	IG: 20 CG: 17	IG: 74.0 CG: 71.8	29.73	N/A	Dementia	Exergame (BrainIT: exergame-based motor-cognitive training)	Home-based	Usual care	≥ 24 minutes, ≥ 5 sessions/ week, 19–24 sessions	Gait speed, IADL
Menengiç et al., 2022 [54]	Turkey	IG: 10 CG: 10	IG: 77.7 CG: 80.6	70.00	18.35	Dementia	DTT (chair-based exercises combined with cognitive tasks)	Telerehabilitation	No intervention	15-40 minutes, 4–5 sessions/week, 6 weeks	TUG, MMSE, FIM
Padala et al., 2017 [55]	United States	IG: 15 CG: 15	IG: 72.1 CG: 73.9	36.67	23.00	Dementia	Exergame (interactive video gaming with physical action)	Home-based	STT	30 minutes, 5 sessions/week, 8 weeks	MMSE, IADL
Salisbury et al., 2024 [56]	United States	IG: 20 CG1: 10 CG2: 8	IG: 69.9 CG1: 75.8 CG2: 72.6	68.42	N/A	MCI	DTT (cycling exercise combined with video game)	Telerehabilitation	STT; Stretching	50 minutes, 3 sessions/week, 12 weeks	Fluid composite score
Swinnen et al., 2023 [57]	Belgium	IG: 7 CG: 11	IG: 81.9 CG: 84.2	94.44	17.20	Dementia	Exergame (VITAAL exergame: stepping movement with cognitive tasks)	Community-based	STT	30 minutes, 3 sessions/week, 12 weeks	1MSTS, MMSE
Trautwein et al., 2020 [58]	Germany	IG: 90 CG: 73	IG: 85 CG: 86	84.05	17.00	Dementia	DTT (strength, balance, endurance, and flexibility training with cognitive tasks)	Community-based	Usual care	60 minutes, 2 sessions/week, 16 weeks	Gait speed
Uğur & Sertel, 2025 [59]	Turkey	IG: 16 CG: 16	IG: 73.75 CG: 73.13	28.13	16.60	Dementia	Exergame (interactive video gaming with physical action)	Community-based	Usual care	30 minutes, 2 sessions/week, 6 weeks	TUG
van Santen et al., 2020 [60]	Netherlands	IG: 73 CG: 39	IG: 79 CG: 79	46.43	18.55	Dementia	Exergame (cycling exercise combined with video game)	Community-based	Usual care	28.33 minutes, 5 sessions/week, 6 months	MMSE
Wu et al., 2025 [61]	China	IG1: 12 IG2: 12 IG3: 12 CG: 12	IG1: 66.00 IG2: 67.14 IG3: 67.29 CG: 66.57	54.17	27.69^a^	MCI	DTT (combined resistance and cognitive exercise)	Community-based	STT	20 minutes, 3 sessions/week, 4 weeks	MoCA
Yu et al., 2025 [62]	China	IG: 36 CG:36	IG: 81.86 CG: 80.75	47.22	23.56^a^	MCI	DTT (combined physical and cognitive exercise)	Community-based	Usual care	20-30 minutes, 4–6 sessions/week, 24 weeks	MoCA

1MSTS, One Minute Sit to Stand Test; 5TSTS, Five Times Sit to Stand Test; CG, Control Group; DTT, Dual-Task Training; FIM, Functional Independence Measure; IADL, Instrumental Activities of Daily Living; IG, Intervention Group; MCE, Mini-Cognitive Examination; MCI, Mild Cognitive Impairment; MMSE, Mini-Mental State Examination; MoCA, Montreal Cognitive Assessment; STT, Single-Task Training; TUG, Timed Up and Go Test.

^a^MMSE-equivalent score derived from the MoCA.

All included studies utilized cognitive-motor DTT. The duration of intervention ranged from 20 to 90 minutes per session (mean 44.78 minutes), 1–6 sessions per week (mean 2.91 weeks), with an overall session of 4–40 weeks (mean 14.87 weeks). This resulted in a total intervention dose ranging from 4 to 60 hours (mean: 27.9 hours). Clinical outcomes included gait speed, functional mobility (Timed Up and Go, One Minute Sit to Stand Test, and Five Times Sit to Stand Test), global cognition (MMSE, MoCA, Mini Cognitive Examination, CogniFit computerized neurocognitive battery, and fluid composite score), and ADL (Barthel Index, Functional Independence Measure, and Instrumented Activity of Daily Living).

### Literature quality

**Fig 2:**
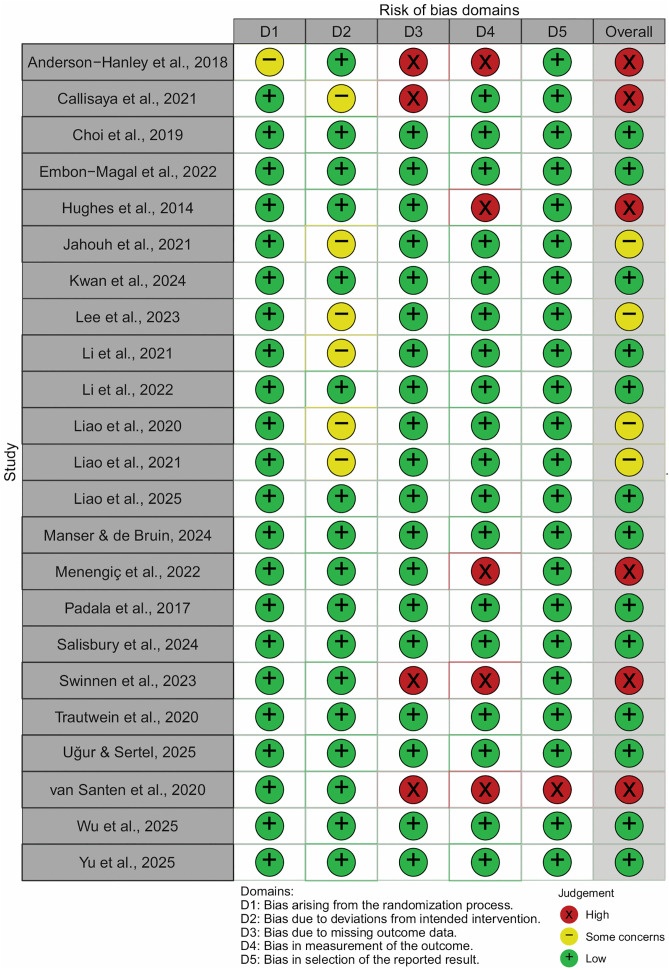
Summary of risk of bias in included studies.

The methodological quality of the included studies assessed by the RoB 2, is presented in **Fig 2,** with details provided in **S3 Table**. The overall risk of bias for each study was rated as “low risk of bias” when all individual domains were judged as low risk; “some concerns” when at least one domain raised some concerns but none were high risk; and “high risk of bias” if at least one domain was judged to be at high risk, or if multiple domains raised some concerns. Thirteen studies [42,43,46,48,49,52,53,55,56,58,59,61,62] were rated as low risk of bias, and four studies [45,47,50,51] were rated as having some concerns. Six studies [40,41,44,54,57,60] were identified as having high risk of bias. Among these, four studies [40,41,57,60] had high risk due to missing outcome data (Domain 3), five studies [40,44,54,57,60] had high risk due to outcome measurement (Domain 4), and one study [60] had high risk due to selective reporting (Domain 5).

### Results of primary outcomes

#### Gait speed.

**Fig 3:**
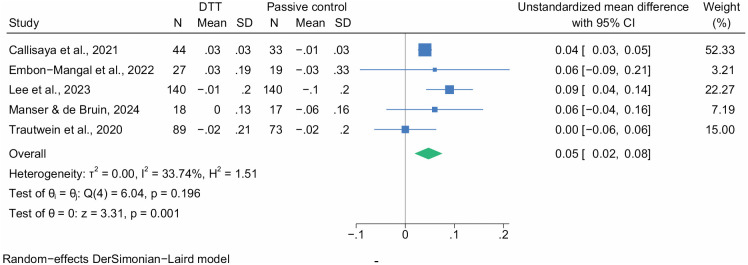
Forest plot of gait speed comparing DTT with passive control.

Seven studies assessed the effectiveness of DTT on gait speed, including two with active control [44,52] and five with passive control [41,43,47,53,58]. When comparing the intervention with passive control, moderate heterogeneity was observed (*I*^2^ = 33.74%, *p* = 0.196). The pooled unstandardized mean difference was 0.05 m/s (95% CI, 0.02–0.08; *z* = 3.31; *p* = 0.001) **(Fig 3)**, indicating that DTT significantly improved gait speed by 0.05 m/s higher than the passive control. However, the network consistency assumption was violated [*χ*^2^(1) = 8.30, *p* = 0.004], which transitivity analysis further revealed that one study [41] delivered the intervention via telerehabilitation, whereas the remaining 6 used home-based or community-based approaches. This discrepancy may have influenced the assumption test. After excluding this study, the network meta-analysis containing 6 studies satisfied the consistency assumption [*χ*^2^(1) = 3.84, *p* = 0.050]. Network meta-analysis indicated that the intervention was likely the most effective in improving gait speed among the three groups (SUCRA = 0.903), with a minimal difference between the active control (SUCRA = 0.297) and passive control (SUCRA = 0.300) groups. For publication bias, Egger’s test revealed no evidence of publication bias. (*t* = -0.66; *p* = 0.536; 95% CI, -1.82–1.07).

#### Functional mobility.

**Fig 4:**
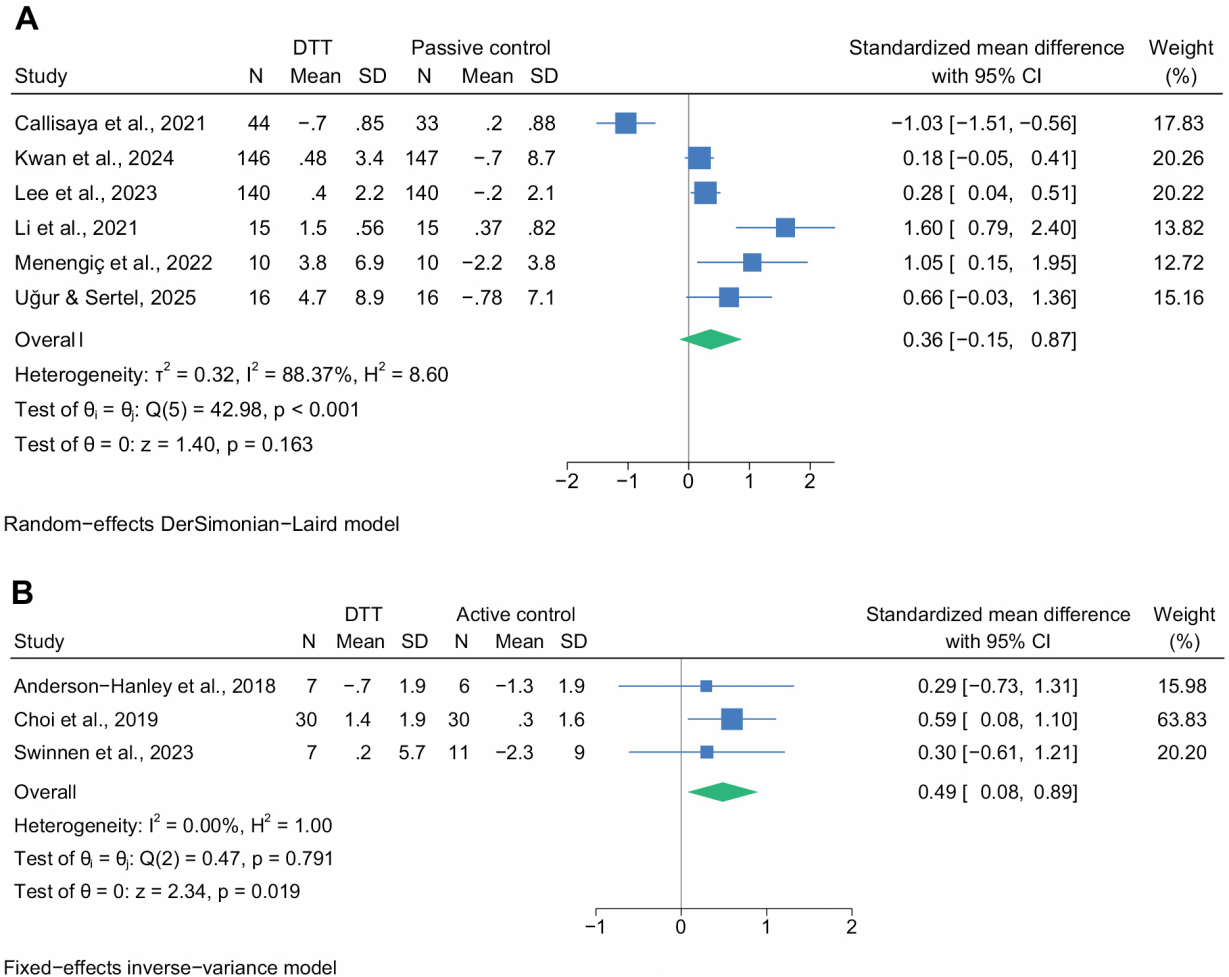
Forest plots of functional mobility. (A) Comparing between DTT and passive control. (B) Comparing between DTT and active control.

Nine studies examined the intervention effect of DTT on functional mobility, with three active controls [40,42,57] and six passive controls [41,46–48,54,59]. Against the passive control, effect sizes were highly heterogeneous across studies (*I*^2^ = 88.37%, *p* < 0.001) and the pooled standardized mean difference was not significant (SMD = 0.36; 95% CI, -0.15– 0.87; *z* = 1.40; *p* = 0.163) **(Fig 4A)**. In contrast, the comparison with the active control showed no heterogeneity (*I*^2^ = 0%, *p* = 0.791) and the pooled standardized mean difference indicated significant improvement (SMD = 0.49; 95% CI, 0.08–0.89; *z* = 2.34; *p* = 0.019) **(Fig 4B)**. All nine studies satisfied the network consistency assumption [*χ*^2^(1) = 1.48, *p* = 0.223]. Network meta-analysis further indicated that the DTT intervention was the most effective at improving global cognition (SUCRA = 0.981), followed by active control (SUCRA = 0.505) and passive control (SUCRA = 0.014). There was no publication bias in functional mobility outcomes (*t* = -0.73; *p* = 0.490; 95% CI, -4.64–2.46).

#### Global cognition.

**Fig 5:**
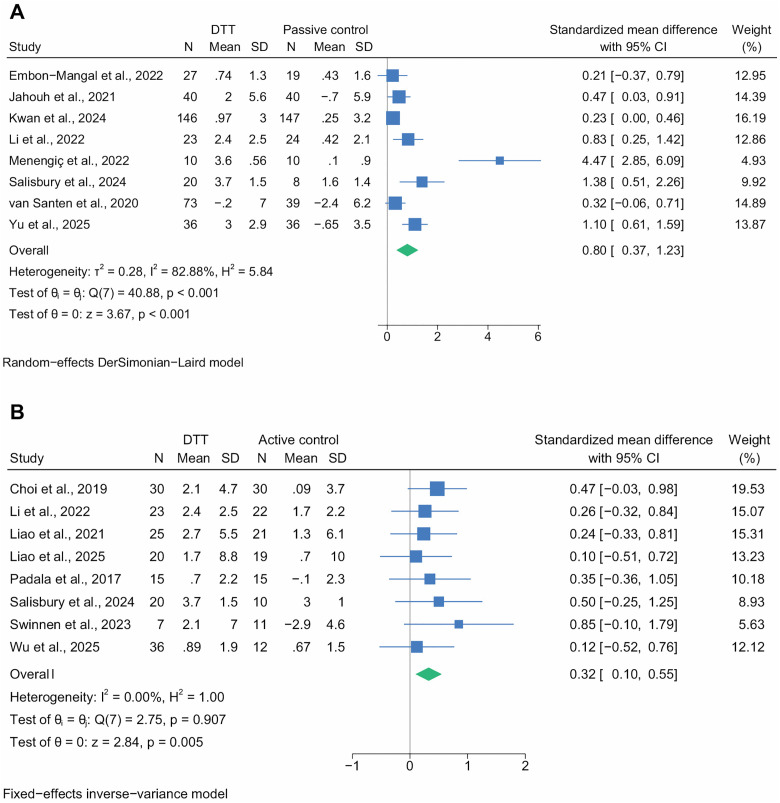
Forest plots of global cognition. (A) Comparing between DTT and passive control. (B) Comparing between DTT and active control.

Fourteen studies examined the effectiveness of DTT on global cognition, including six studies with active control [42,51,52,55,57,61], six studies with passive control [43,45,46,54,60,62], and two with both active and passive controls [49,56]. When comparing with the passive control, high heterogeneity was observed (*I*^2^ = 82.88%, *p* < 0.001); however, pooled standardized mean difference showed that there was significant improvement (SMD = 0.80; 95% CI, 0.37–1.23; *z* = 3.67; *p* < 0.001) **(Fig 5A)**. On the other hand, no heterogeneity was shown when comparing with active control (*I*^2^ = 0%, *p* = 0.907) with pooled standardized mean difference indicating significant improvement (SMD = 0.32; 95% CI, 0.10–0.55; *z* = 2.84; *p* = 0.005) **(Fig 5B)**. All fourteen studies satisfied the network consistency assumption [*χ*^2^(2) = 2.47, *p* = 0.291], which network meta-analysis further indicated that the intervention group was the most effective at improving global cognition (SUCRA = 0.981), followed by active control (SUCRA = 0.505) and passive control (SUCRA = 0.014). Nevertheless, statistical analysis indicated publication bias in this outcome (*t* = -3.02; *p* = 0.008; 95% CI, -3.85–-0.67).

#### ADL.

**Fig 6:**
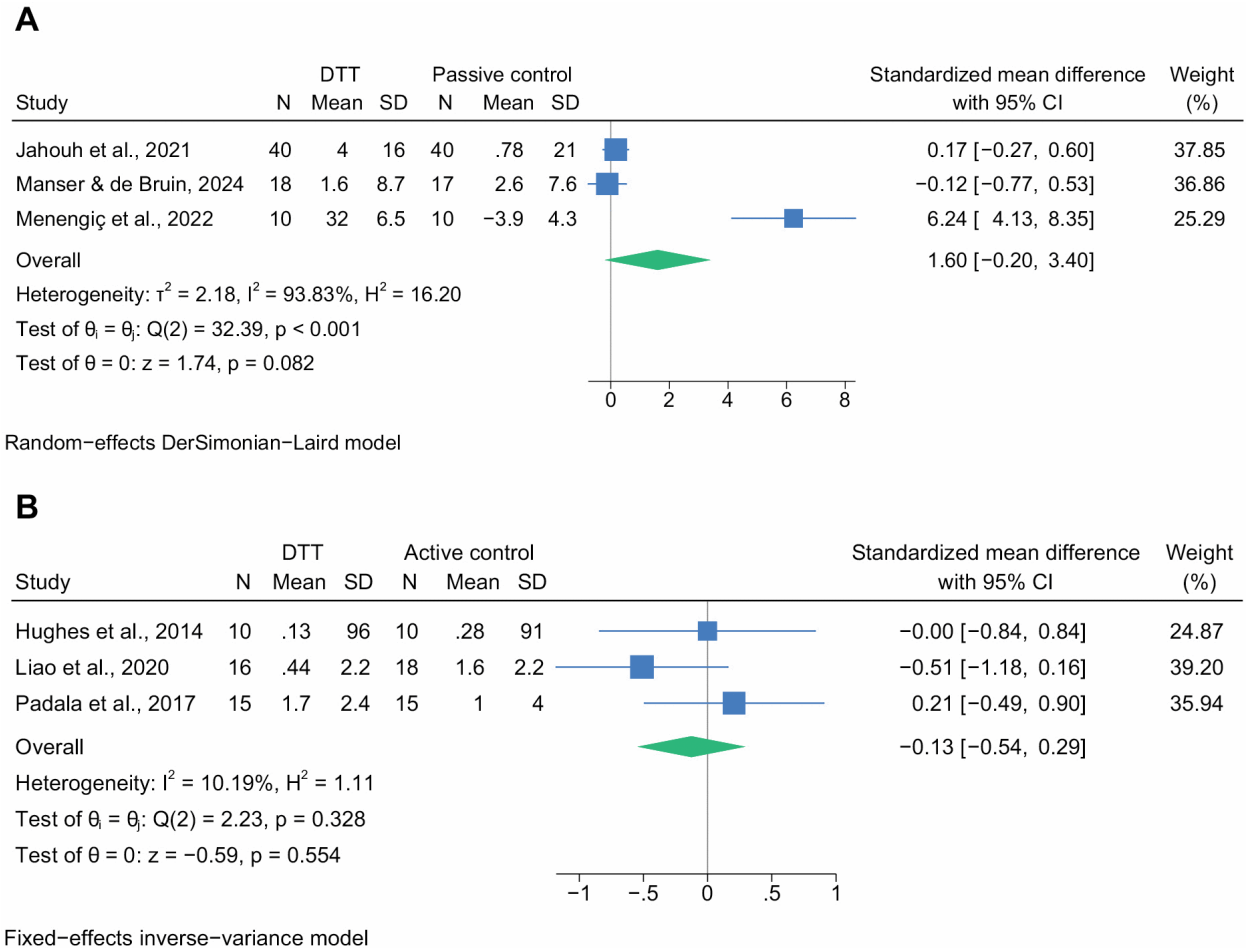
Forest plots of ADL. (A) Comparing between DTT and passive control. (B) Comparing between DTT and active control.

Six studies examined the effectiveness of DTT on ADL, with three against active control [44,50,55] and three against passive control [45,53,54]. Heterogeneity was observed among studies comparing interventions with passive controls (*I*^2^ = 93.83%, *p* < 0.001), with no difference in effect between groups (SMD = 1.60; 95% CI, -0.20–3.40; *z* = 1.74; *p* = 0.082) **(Fig 6A)**. On the other hand, comparing with active control, homogeneity across studies was observed (*I*^2^ = 10.19%, *p* = 0.328); however, standardized mean difference revealed non-significant difference between groups (SMD = -0.13; 95% CI, -0.54–0.29; *z* = -0.59; *p* = 0.554) **(Fig 6B)**. All 6 studies met the network consistency assumption [*χ*^2^(1) = 1.75, *p* = 0.186], and NMA revealed that active control was most likely to yield the best outcome for ADL (SUCRA = 0.694), although it was closely followed by intervention (SUCRA = 0.687), and passive control ranked the lowest (SUCRA = 0.120). No publication bias was shown in ADL according to statistical analysis (*t* = -1.89; *p* = 0.132; 95% CI, -10.35–1.98).

**Fig 7:**
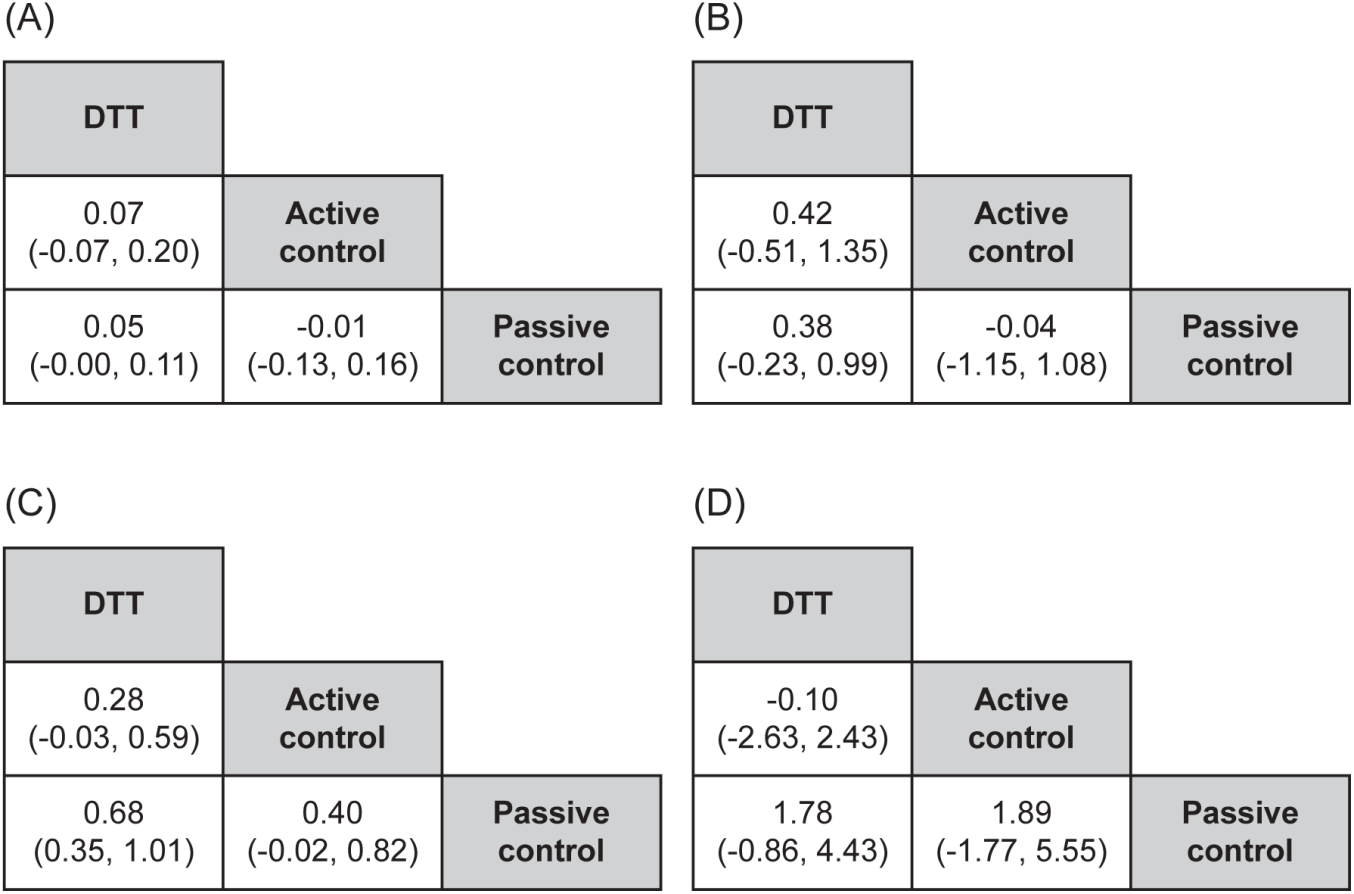
League table presenting effect estimates. (A) of gait speed. (B) functional mobility. (C) global cognition. (D) ADL.

Results of network meta-analysis, including network map and ranking curves, and comparison-adjusted plots for publication bias, are shown in **S1 Fig**. Effect estimates for all pairwise comparisons are presented in the league tables **(Fig 7)**, with each cell showing a comparison of the row-defining treatment against the column-defining treatment. Positive values (> 0) indicate a benefit favoring the column-defining treatment, whereas negative values (< 0) indicate a benefit favoring the row-defining treatment. **Fig 7A** shows unstandardized mean difference of gait speed, **Fig 7B** shows standardized mean difference of functional mobility, **Fig 7C** shows standardized mean difference of global cognition, and **Fig 7D** shows standardized mean difference of ADL.

### Meta-regression and subgroup analyses

Pairwise meta-analyses revealed heterogeneity in all four outcomes when comparing the intervention to the passive control, leading to meta-regression analysis as its result shown in **Table 2**. For gait speed, geographical region, percentage of females, intervention type, and total weeks of intervention decreased the τ² value by more than 50%, suggesting these are potential sources of heterogeneity. Geographical region was also identified as a potential source for functional mobility. In contrast, no covariates were identified as sources of heterogeneity for global cognition. Finally, although meta-regression indicated that intervention type and mode might contribute to heterogeneity for ADL, the limited number of studies prevented further subgroup analysis.

**Table 2:** Results of meta-regression analysis.

Covariate	Empty model			Residual heterogeneity after fitting covariate		
	N	τ²	*I* ^2^	N	τ²	*I* ^2^
**Gait speed (intervention vs passive control)**						
Geographical region	5	0.00034	33.74	5	0*	0
Age	5	0.00034	33.74	5	0.00083	48.11
Percentage of female	5	0.00034	33.74	5	0*	0
Intervention type	5	0.00034	33.74	5	0*	0
Intervention mode	5	0.00034	33.74	5	0.002069	60.88
Duration per session	5	0.00034	33.74	5	0.000474	26.12
Frequency	5	0.00034	33.74	5	0.000749	35.75
Total weeks	5	0.00034	33.74	5	0*	0
Baseline MMSE	3	0	0	3	0*	0
**Functional mobility (intervention vs passive control)**						
Geographical region	6	0.319	88.37	6	0*	0
Age	6	0.319	88.37	6	0.2568	84.57
Percentage of female	6	0.319	88.37	6	0.6876	90.66
Intervention type	6	0.319	88.37	6	0.3668	89.98
Intervention mode	6	0.319	88.37	6	0.3668	89.98
Duration per session	6	0.319	88.37	6	0.4112	89.16
Frequency	6	0.319	88.37	6	0.2833	86.44
Total weeks	6	0.319	88.37	6	0.7233	90.65
Baseline MMSE	5	0.6839	90.51	5	0.4707	87.73
**Global cognition (intervention vs passive control)**						
Geographical region	8	0.2798	82.88	8	0.4024	85.76
Age	8	0.2798	82.88	8	0.3883	84.75
Percentage of female	8	0.2798	82.88	8	0.4078	83.85
Intervention type	8	0.2798	82.88	8	0.2579	80.93
Intervention mode	8	0.2798	82.88	8	0.1996	78.29
Duration per session	8	0.2798	82.88	8	0.3487	82.69
Frequency	8	0.2798	82.88	8	0.2633	79.91
Total weeks	8	0.2798	82.88	8	0.3519	84.08
Baseline MMSE	7	0.2663	83.6	7	0.3735	86.22
**ADL (intervention vs passive control)**						
Geographical region	3	2.182	93.83	3	2.182	93.83
Age	3	2.182	93.83	3	24.74	96.89
Percentage of female	3	2.182	93.83	3	9.443	96.29
Intervention type	3	2.182	93.83	3	0*	0
Intervention mode	3	2.182	93.83	3	0*	0
Duration per session	3	2.182	93.83	3	22.75	96.9
Frequency	3	2.182	93.83	3	22.91	96.91
Total weeks	3	2.182	93.83	3	9.663	96.31
Baseline MMSE	2	17.82	96.73	2	13.89	100

MMSE, Mini-Mental State Examination; N, Number of studies.

* τ² reduced greater than 50%.

Subgroup analysis further explored these findings. For gait speed (DTT vs. passive control), significant subgroup effects were observed for intervention type [Q(1) = 4.15, *p* = 0.042] and percentage of female participants (< 50% vs. ≥ 50%) [Q(1) = 4.15, *p* = 0.042]. In contrast, no significant subgroup effects were observed for the intervention mode [Q(1) = 0.24, *p* = 0.889] or type of cognitive impairment [Q(1) = 1.15, *p* = 0.283]. For functional mobility (DTT vs. passive control), no significant subgroup effects were found for type of cognitive impairment [Q(1) = 2.23, *p* = 0.135], type of DTT [Q(1) = 0.08, *p* = 0.777], or intervention mode [Q(1) = 0.08, *p* = 0.777]. For global cognition, subgroup analyses revealed no significant effects across variables. This included type of cognitive impairment [DTT vs. active control: Q(1) = 0.58, *p* = 0.447; DTT vs. passive control: Q(1) = 0.05, *p* = 0.829], baseline cognitive impairment severity [DTT vs. active control: Q(2) = 1.59, *p* = 0.451; DTT vs. passive control: Q(1) = 0.73, *p* = 0.393], type of DTT [DTT vs. active control: Q(1) = 0.71, *p* = 0.399; DTT vs. passive control: Q(1) = 3.06, *p* = 0.080], and intervention mode [DTT vs. active control: Q(2) = 0.02, *p* = 0.988; DTT vs. passive control: Q(1) = 3.74, *p* = 0.053]. The details of the subgroup analyses are shown in **S2 Fig**.

### Results of secondary outcomes

#### Adverse events.

Four out of 23 studies reported adverse events related to nonclinic-based DTT [41,46,49,57]. The most common adverse events were musculoskeletal pain or discomfort (10 events). Other events were hernia-related pain (1 event), episodes of vertigo or fainting (2 events), and a noninjurious fall (1 event). All adverse events were mild in severity and did not require hospitalization.

#### Feasibility.

Eleven out of 23 studies reported adherence rates, with an average adherence rate of 80.82% [41–43,46,48,49,53,55–58]. Some participants with cognitive impairment reported difficulties during both the intervention and assessment, requiring extensive verbal guidance [41,56,57]. In addition, technical barriers, including unstable internet connections or limited access to necessary devices, were reported in two studies [49,53], with one attributing these issues as a primary reason for nonadherence [53].

#### Acceptability.

Eleven out of 23 studies reported positive feedback from participants, indicating that nonclinic-based DTT was comfortable, enjoyable, interesting, well-tolerated, beneficial, and satisfactory [41–44,46,48,49,53,54,57,61]. Nevertheless, some studies reported that the intervention became repetitive over time, leading to reduced interest, motivation, and adherence [41,50,55].

#### Caregiver burden.

Caregiver-related factors were also influential, as one study reported that participants dropped out due to caregiver unavailability [55]. Another study noted that caregivers experienced increased burdens during the COVID-19 lockdown, as they had to spend more time supporting [54].

## Discussion

### Gait speed, functional mobility, global cognition, and ADL

Nonclinic-based DTT significantly improved gait speed, functional mobility, and global cognition in older adults with cognitive impairment. These findings are consistent with previous evidence from healthy older adults and neurological populations, including stroke, PD, and MS [65–72]. Given the established association between mobility and cognition, improvements in gait and functional mobility may be associated with enhanced global cognition, and vice versa [73,74].

Several mechanisms may explain these benefits. DTT simultaneously engages motor and cognitive processes, thereby more closely reflecting the demands of everyday activities than STT. Physical exercise within DTT may promote neural plasticity, increase brain-derived neurotrophic factor production, enhance energy metabolism, and reduce glial activation and neuroinflammation, all of which support cognitive function [15,16,75]. In addition, the integration of cognitive challenges with motor tasks may improve attentional allocation, task switching, balance control, and gait automaticity [76–82]. Enhanced gait automaticity may reduce reliance on conscious executive control during walking, thereby facilitating more efficient gait performance and contribute to improvements in gait speed and functional mobility [70,83].

Beyond these neurophysiological effects, DTT may offer greater transferability to everyday activities than STT. Real-world activities rarely occur in isolation and typically require the simultaneous coordination of motor and cognitive processes. Therefore, engaging in physical exercise alone may not be sufficient to produce meaningful cognitive improvements. This concept is supported by a study involving individuals with idiopathic normal pressure hydrocephalus, in which STT focused solely on gait and balance failed to produce significant cognitive improvements following the intervention [84]. These findings underscore that effective cognitive enhancement may require training programs that integrate cognitive challenges with physical tasks, thereby more closely reflecting the complex demands of daily life.

While the benefits of DTT for physical and cognitive function were evident, substantial heterogeneity was observed across studies, and the sources of variability differed according to the outcome examined. A major contributor appears to be the nature of the control conditions. For both functional mobility and global cognition, comparisons against passive controls exhibited substantial heterogeneity that could not be explained by the covariates examined in this review. Notably, for functional mobility, this variability obscured any significant difference between DTT and passive controls despite DTT significantly outperforming active controls. One possible explanation is the considerable variation among passive control conditions, which ranged from no intervention to stretching exercises and standard usual care. Methodological research has demonstrated that the term “usual care” encompasses a broad spectrum of clinical practices, potentially reducing measurable differences between study groups and obscuring the true effect size of an intervention [85].

In contrast, the heterogeneity observed in gait speed outcomes was partially explained by intervention and participant characteristics. Subgroup analyses demonstrated that exergaming-based DTT yielded greater improvements in gait speed than other forms of DTT. This finding is consistent with previous research showing that VR-based training improves gait performance more effectively than traditional DTT [63]. The enhanced efficacy of exergaming may be attributable to its ability to provide real-time feedback and immersive environments that continuously challenge visual attention and motor adaptation, features that are often less prominent in conventional DTT programs. Another factor contributing to gait speed heterogeneity was sex composition, with studies including a lower proportion of female participants reporting greater improvements in gait speed. This finding may be related to sex-related differences in balance and gait characteristics, as older males generally demonstrate greater postural stability and more stable gait patterns than older females [86]. Additionally, males tend to exhibit wider step widths and longer step lengths [87]. These biomechanical advantages may facilitate more effective engagement in technology-assisted DTT, which often requires dynamic weight shifting and multidirectional stepping, thereby contributing to larger gait speed improvements.

Interpreting the benefits of DTT on cognition is complex due to the variety of measurements available. Although global cognition measured using MMSE or MoCA consistently improved, the high heterogeneity against passive controls remains unexplained. Despite concerns regarding the ceiling effects of these assessment tools [88], they remain robust indicators of multi-domain cognitive function. However, inconsistent reporting of cognitive outcomes across the primary studies prevented domain-specific analyses (e.g., executive function or memory), limiting the ability to determine which cognitive domains were most responsive to DTT.

The lack of significant improvement in ADL may reflect the complex and multifactorial nature of functional independence rather than the limited effects of DTT. ADL requires the integration of multiple domains, including executive function, attention, motor control, environmental adaptation, and task-specific skills, which may not be fully captured by improvements in isolated clinical outcomes. This limited translation may be explained by the gap between structured training environments and real-world performance. ADL is typically performed in dynamic and unpredictable contexts requiring continuous cognitive-motor integration, which may not be fully replicated in DTT protocols [89]. In addition, many DTT programs primarily target general cognitive-motor capacity rather than direct practice of daily functional tasks, which may further limit transfer to functional independence.

### Implementation and utilization in nonclinical settings

This review highlighted a significant shift toward technology-aided rehabilitation in nonclinical settings, with most included studies (20 out of 23 studies) incorporating digital technologies into their interventions. Technology-supported approaches ranged from immersive virtual environments and VR-based exercise systems to commercially available motion-capture platforms such as Nintendo Wii Fit and Microsoft Kinect. The widespread adoption of these technologies suggests increasing recognition of their potential to enhance accessibility, engagement, and delivery of rehabilitation outside traditional clinical environments.

Overall, nonclinic-based DTT demonstrated a favorable safety profile. Reported adverse events were generally mild and infrequent, with musculoskeletal discomfort being the most common complaint, and no serious adverse events requiring hospitalization were reported. These findings indicate that DTT can be safely implemented in home and community settings when appropriate precautions and monitoring are in place.

The interventions also demonstrated high feasibility and acceptability. Participants frequently described the programs as enjoyable, comfortable, and beneficial, and adherence rates were consistently high across studies. These findings are consistent with previous evidence suggesting that the engaging and interactive nature of technology-supported interventions may promote long-term participation and adherence, particularly when gamified elements are incorporated [90]. Despite these positive findings, there are some challenges affecting participation, including cognitive limitations, technical difficulties, repetitive training tasks, and caregiver-related constraints. Similar challenges have been reported in studies involving individuals with PD and older adults with and without chronic conditions [91–93]. These findings highlight the importance of participant screening, tailored intervention design, caregiver support, user-friendly technologies, and clear instructions, which may help maximize engagement, adherence, and safety.

## Strengths and limitations

This review is the first to specifically examine nonclinic-based DTT interventions in older adults with cognitive decline. By focusing on home- and community-based approaches, it provides evidence that individuals with limited access to healthcare facilities may still benefit from interventions targeting both physical and cognitive function. Additional strengths include the use of both pairwise and network meta-analyses, comprehensive literature searches, and systematic risk-of-bias assessment using the Cochrane RoB 2 tool in accordance with PRISMA guidelines.

However, several limitations should be considered when interpreting these findings. First, although most included studies were rated as having a low risk of bias or some concerns, missing outcome data and lack of assessor blinding were common sources of methodological bias. Statistical tests also suggested potential publication bias for global cognition. This finding should be interpreted cautiously, as one study reported a substantially larger effect size than the others; therefore, the observed funnel plot asymmetry may reflect between-study heterogeneity or a small-study effect rather than true publication bias. Publication bias related to secondary outcomes, particularly adverse events and participant attrition, can also not be entirely ruled out. Although grey literature and clinical trial databases were searched to minimize this risk, unpublished studies with less favorable safety findings may not have been captured. Second, analyses were limited by the availability and consistency of reported data. The small number of studies reporting ADL precluded more comprehensive meta-analyses and subgroup analyses. Similarly, inconsistent reporting of cognitive outcomes prevented analyses of specific cognitive domains, and the meta-analysis of gait speed was restricted to comparisons with passive controls because of the limited number of studies using active controls. Finally, most included studies evaluated outcomes immediately after the intervention. The limited availability of follow-up data restricts conclusions regarding the long-term sustainability of the observed benefits.

## Conclusions

Overall, our results support the idea that nonclinic-based DTT improves physical and cognitive functions, including gait speed, functional mobility, and global cognition, among older adults with cognitive decline. These results highlight the feasibility and effectiveness of delivering DTT outside traditional clinical settings, offering a valuable complement to in-clinic rehabilitation. By implementing interventions in nonclinical environments, the burden of frequent hospital or clinic visits, including travel costs, healthcare expenses, and accessibility barriers, can be reduced, thereby facilitating greater participant engagement, enhancing adherence, and promoting sustained involvement in training. Successful implementation requires appropriate preparation of equipment, reliable communication systems, and caregiver support to ensure continuity of training and maintain participant motivation. Overall, nonclinic-based DTT represents a pragmatic, participant-centered approach to supporting functional independence in this population, and technology plays a pivotal role in delivering rehabilitation directly to patients’ residences.

## Supporting information

S1 Checklist PRISMA checklist. PRISMA checklist.
(DOCX)

S1 Table Search strategy and search results for each database. (DOCX)

S2 Table Excluded studies in screening processes and reasons for exclusion. (XLSX)

S3 Table Risk of bias assessment of included studies. (XLSX)

S1 Data Dataset for meta-analysis. (XLSX)

S1 Fig Results of network meta-analysis and publication bias. (DOCX)

S2 Fig Results of subgroup analysis. (DOCX)
