# Supplementary material for: Effectiveness of nonclinic-based dual-task training on physical and cognitive functions and activities of daily living in older adults with cognitive decline: A systematic review and meta-analysis
Source: PLOS Digit Health. 2026 Aug 6;5(8):e0001571. doi: 10.1371/journal.pdig.0001571 (PMC13446693; doi:10.1371/journal.pdig.0001571)
Supplement: Supplementary file 2 [file pdig.0001571.s002.docx]

**Search strategy and search results for each database**

| **Database** | **Search strategy** | **Results** |
| --- | --- | --- |
| PubMed | (Cognitive dysfunction[MeSH] OR “cognitive declin*” OR “cognitive impairment*” OR Dementia[MeSH] OR Alzheimer*) AND (Video games[MeSH] OR videogam* OR “video gam*” OR “exergam*” OR Nintendo OR Wii OR Xbox OR Kinect OR Virtual Reality[MeSH] OR “virtual realit*” OR dual-task OR cognitive-motor OR motor-cognitive OR motor-motor) AND (tele* OR telemedicine OR home OR synchronous OR remot* OR web OR interactive OR phone OR “video call*” OR online) | 785 |
| Embase | (('cognitive defect'/exp OR 'cognitive defect') OR 'cognitive declin*' OR 'cognitive impairment*' OR 'dementia' OR ('alzheimer disease'/exp OR 'alzheimer*')) AND ((('tele*' OR 'telemedicine') OR ('video consultation'/exp OR 'video consultation') OR home OR synchronous OR remot* OR web OR interactive OR phone OR 'video call*' OR online) AND ((('video game'/exp OR 'video gam*') OR 'videogam*' OR 'exergam*' OR nintendo OR wii OR xbox OR kinect OR 'virtual realit*') OR ('dual task' OR 'motor-cognitive' OR 'cognitive-motor' OR 'motor-motor'))) | 1,203 |
| CINAHL | ((MH “Cognition Disorders+”) OR (MH “Alzheimer’s Disease”) OR (MH “Dementia+”) OR “cognitive impairment*” OR “cognitive declin*”) AND ((MH “Video Games+”) OR “video gam*” OR “videogam*” OR exergam* OR Nintendo OR Wii OR Xbox OR Kinect OR (MH “Virtual Reality+”) OR “virtual realit*” OR “dual-task” OR “motor-cognitive” OR “cognitive-motor” OR “motor-motor”) AND ((MH “Telemedicine+” OR Tele* OR home OR synchronous OR remot* OR web OR interactive OR phone OR “video call*” OR online) | 226 |
| Cochrane Central Register of Controlled Trials (CENTRAL) | 1 \| Cognitive dysfunction.sh.; 2 \| Cognitive declin*.af.; 3 \| Cognitive impairment*.af.; 4 \| Dementia.sh.; 5 \| Alzheimer*.af.; 6 \| 1 or 2 or 3 or 4 or 5; 7 \| Video games.sh.; 8 \| videogam*.af.; 9 \| video gam*.af.; 10 \| exergam*.af.; 11 \| Nintendo.af.; 12 \| Wii.af.; 13 \| Xbox.af.; 14 \| Kinect.af.; 15 \| virtual realit*.af.; 16 \| dual-task.af.; 17 \| cognitive-motor.af.; 18 \| motor-cognitive.af.; 19 \| motor-motor.af.; 20 \| 7 or 8 or 9 or 10 or 11 or 12 or 13 or 14 or 15 or 16 or 17 or 18 or 19; 21 \| tele*.af.; 22 \| home.af.; 23 \| synchronous.af.; 24 \| remot*.af.; 25 \| web.af.; 26 \| interactive.af.; 27 \| phone.af.; 28 \| video call*.af.; 29 \| online.af.; 30 \| 21 or 22 or 23 or 24 or 25 or 26 or 27 or 28 or 29; 31 \| 20 and 30; 32 \| 6 and 31 | 315 |
| IEEE Xplore | ("cognitive impairment" OR "cognitive impairments" OR "cognitive decline" OR "Alzheimer" OR "Alzheimer's" OR "Dementia") AND ("video gam*" OR "videogam*" OR "video-gam*" OR exergam* OR Nintendo OR Wii OR Xbox OR Kinect OR "virtual realit*" OR "dual-task" OR "cognitive-motor" OR "motor-cognitive" OR "motor-motor") AND (tele* OR telemedicine OR home OR synchronous OR remot* OR web OR interactive OR phone OR "video call*" OR online) | 197 |
| ProQuest (theses and dissertations) | ("cognitive impairment*" OR "cognitive declin*" OR dementia OR Alzheimer*) AND ((videogam* OR "videogame*" OR "video-gam*" OR exergam* OR Nintendo OR Wii OR Xbox OR Kinect OR "virtual realit*" OR "dual-task" OR "cognitive-motor" OR "motor-cognitive" OR "motor-motor") AND (tele* OR home OR synchronous OR remot* OR web OR interactive OR phone OR "video call*" OR online)) AND YR(>2013) | 1,121 |
