# Supplementary material for: Effectiveness of nonclinic-based dual-task training on physical and cognitive functions and activities of daily living in older adults with cognitive decline: A systematic review and meta-analysis
Source: PLOS Digit Health. 2026 Aug 6;5(8):e0001571. doi: 10.1371/journal.pdig.0001571 (PMC13446693; doi:10.1371/journal.pdig.0001571)
Supplement: Supplementary file 3 [file pdig.0001571.s006.docx]

**Results of network meta-analysis and publication bias**

**Fig A.** **Network plots for each outcome measure: (a) gait speed, (b) functional mobility, (c) global cognition, and (d) ADL.**

**Fig B.** **The SUCRA rank test for gait speed among the treatment groups.** (Intervention in this plot refers to DTT)

**Fig C.** **The SUCRA rank test for functional mobility among the treatment groups.** (Intervention in this plot refers to DTT)

**Fig D. The SUCRA rank test for global cognition among the treatment groups.** (Intervention in this plot refers to DTT)

**Fig E.** **The SUCRA rank test for ADL among the treatment groups.** (Intervention in this plot refers to DTT)

**Fig F. Comparison-adjusted funnel plots for (a) gait speed, (b) functional mobility, (c) global cognition, and (d) ADL.**
