# Supplementary material for: Effectiveness of nonclinic-based dual-task training on physical and cognitive functions and activities of daily living in older adults with cognitive decline: A systematic review and meta-analysis
Source: PLOS Digit Health. 2026 Aug 6;5(8):e0001571. doi: 10.1371/journal.pdig.0001571 (PMC13446693; doi:10.1371/journal.pdig.0001571)
Supplement: Supplementary file 4 [file pdig.0001571.s007.docx]

**Results of subgroup analysis**

**Fig A. Subgroup analysis of gait speed comparing DTT with passive control, by type of cognitive impairment.**

**Fig B. Subgroup analysis of gait speed comparing DTT with passive control, by percentage of female.**

**Fig C. Subgroup analysis of gait speed comparing DTT with passive control, by type of DTT.**

**Fig D. Subgroup analysis of gait speed comparing DTT with passive control, subgroup by mode of intervention.**

**Fig E. Subgroup analysis of functional mobility comparing DTT with passive control, subgroup by type of cognitive impairment.**

**Fig F. Subgroup analysis of functional mobility comparing DTT with passive control, subgroup by type of DTT.**

**Fig G. Subgroup analysis of functional mobility comparing DTT with passive control, subgroup by mode of intervention.**

**Fig H. Subgroup analysis of global cognition comparing DTT with passive control, subgroup by type of cognitive impairment.**

**Fig I. Subgroup analysis of global cognition comparing DTT with passive control, subgroup by severity of cognitive impairment.**

**Fig J. Subgroup analysis of global cognition comparing DTT with passive control, subgroup by type of DTT.**

**Fig K. Subgroup analysis of global cognition comparing DTT with passive control, subgroup by mode of intervention.**

**Fig L. Subgroup analysis of global cognition comparing DTT with active control, subgroup by type of cognitive impairment.**

**Fig M. Subgroup analysis of global cognition comparing DTT with active control, subgroup by severity of cognitive impairment.**

**Fig N. Subgroup analysis of global cognition comparing DTT with active control, subgroup by type of DTT.**

**Fig O. Subgroup analysis of global cognition comparing DTT with active control, subgroup by mode of intervention.**
